# The Relationship between Trail Running Withdrawals and Race Topography

**DOI:** 10.3390/sports5040091

**Published:** 2017-12-05

**Authors:** Antonini Philippe Roberta, Rochat Nadège, Crettaz Von Roten Fabienne, Hauw Denis

**Affiliations:** 1 Institute of Sport Sciences, University of Lausanne, Géopolis, 1015 Lausanne, Switzerland; nadege.rochat@unil.ch (R.N.); Fabienne.crettazvonroten@unil.ch (C.V.R.F.); Denis.hauw@unil.ch (H.D.); 2 CETAPS Laboratory, Faculty of Sports Sciences, University of Rouen, EA 3832 Rouen, France

**Keywords:** experience, topography, ultra-endurance, course of action, situated action, meanings

## Abstract

**Context:** A growing amount of recent research in sport psychology has focused on trying to understand withdrawals from ultra-races. However, according to the Four E approach, the studies underestimated the embedded components of these experiences and particularly how they were linked to the specific environmental conditions in which the experiences occurred. **Objective**: This study aimed to characterize trail running withdrawals in relationship to race topography. **Design**: Qualitative design, involving self-confrontation interviews and use of a race map. **Setting**: Use of the race map for description of the race activity and self-confrontation interviews took place 1–3 days after the races. **Participants**: Ten runners who withdrew during an ultra-trail race. **Data Collection and Analysis**: Data on past activity traces and experiences were elicited from self-confrontation interviews. Data were coded and compared to identify common sequences and then each type of sequence was counted with regard to race topography. **Results**: Results showed that each sequence was related to runners’ particular possibilities for acting, feeling, and thinking, which were in turn embedded in the race topography. These sequences allowed the unfolding of the activity and increased its overall effectiveness in relation to the constraints of this specific sport. **Conclusion**: This study allowed us to highlight important information on how ultra-trail runners manage their races in relationship to the race environment and more specifically to its topography. The result will also help us to recommend potential adjustments to ultra-trail runners’ performance-oriented training and preparation.

## 1. Introduction

Ultra-trail races involve running semi-autonomously for more than 80 km along marked trails in natural environments. Such races are reputed to be exhausting, requiring runners to push themselves to the limits of their endurance [1]. They can be considered as extreme sports or dangerous activities. Many participants are unable to find the reserves of energy needed to finish races, and it is thus not surprising that event statistics report high proportions of withdrawals during races. For example, 36% and 48% of participants were non-finishers in the Ultra Trail Mont Blanc (UTMB) 2015 and the Grand Raid 2014 on the island of Réunion, respectively [2]. The inherent difficulties of an ultra-trail race mean that participants must find, draw on, and make the most effective use of their physiological and psychological energy reserves.

A growing amount of recent research in sport psychology has focused on trying to understand withdrawals from such ultra-races [3,4,5,6,7]. Two distinct bodies of research can be identified. The first focuses on ultra-race running at a general level and reveals the different psychological processes involved in performance. Several authors have shown that finishing is linked to strategies for the regulation of emotions, pain, and negative energy balances [5,8,9]. The conclusion here is that these key psychological factors are also involved in avoiding withdrawal. For example, runners used coping strategies to deal with problems concerning food and hydration, as well as mental techniques such as dissociation and goal setting [5]. Lahart et al. [9] studied emotions, the perceived functionality of emotions, and strategies for regulating them. However, the limitation of these approaches is the lack of insights into the entire activity of runners. Aiming to better understand trail runners’ activity with a holistic perspective, Hauw et al. [4] conducted a situated analysis of how runners experience their race situations and were able to finish the race successfully. The results showed various ways of experiencing and managing the different types of efforts required to complete an ultra-trail. These were described as “preserving oneself”, “running without discomfort”, “running with sudden extreme fatigue”, “running at the best possible pace”, “controlling the pace as the race unfolds”, and “accelerating to finish the race”. Here, the runners’ pattern of experience described how they managed the entire activity in order to finish the race, attain the best possible ranking, or even try to win. 

The second body of research has focused directly on the withdrawals per se. Rochat et al. [6] characterized ultra-trail running athletes’ experiences as constituted by the dynamics of the emergence of different vitality states, such as a state of vitalty preservation (SVP), a state of vitality loss (SVL), and a state of vitality revival (SVR). They then compared the distribution and dynamics of the vitality states experienced by both finishers and withdrawers during their race. Results showed that withdrawers had significantly fewer sequences of SVP than finishers, but also significantly more sequences of SVL. Furthermore, the authors observed a significant difference in the emergence of SVP in finishers in comparison to withdrawers from the second quarter of the race, and there was a significant emergence of SVL in withdrawers after the third quarter of the race. In addition, withdrawers tended to remain in an SVL, whereas finishers were better able to exit SVL by trying to preserve themselves when they felt their body or mind giving them a physiological or psychological warning sign. The results showed the importance of maintaining an SVP in this kind of race, particularly at the beginning, and of adapting one’s running activity after a difficult section of trail in order to seek relative physical preservation and the ability to complete the race. Focusing on a qualitative understanding of withdrawals, Antonini Philippe et al. [3] analysed the experience of ten runners who withdrew during the ultra-trail race on the island of Réunion. Using self-confrontation interviews, they reconstructed the dynamics of their successive psychological states and the activities that led to withdrawal. They identified seven representative sequences: (a) feeling pain; (b) putting meaning to those feelings; (c) adjusting running style; (d) attempting to overcome the problem; (e) the influence of other runners; (f) assessing the situation; and (g) deciding to withdraw. The study revealed a characteristic or representative story of withdrawal that could be described as a progressive, cumulative process associating diverse bodily, behavioural, cognitive, and social experiences. 

The originality of these two bodies of research is their concern about the analysis of runners’ experiences in situ. Most of these recent studies used the theoretical framework and methodology of course of experience theory [10]. This is part of the well-known Four E approach to activity (embodied, embedded, experienced, and enacted) that has been labeled as a new wave in cognitive science [11,12,13,14] and has recently been explicitely displayed in sport psychology [6,15]. The Four E approach conceives human activity as being (a) embedded in the dynamics of the changing situation [12]; (b) extended by tools or cultural artifacts [16]; (c) embodied as recurrent perception-action patterns [17,18]; and (d) enacted, meaning that a person brings forth his/her own world through his/her specific asymmetrical relationships with his/her environment [11,14].

With a particular focus on the phenomenological level of sporting performance, these studies hypothesized that an analysis of the different streams of experience meaningful to each person, i.e., what is “showable, narratable and commentable to an observer or interlocutor” [19], is well-suited to understanding the psychological organization of performance when using the fundamental components of the Four E properties of activity. Hence, these studies made additional contributions to the research that has used this approach to show how perceived experiences during a sporting activity could be thought of as a succession of discrete sequences that have an impact on the outcome of that activity [20,21,22,23,24,25,26]. However, according to the 4 E approach, the studies underestimated the embedded components of these experiences, and particularly how they were linked to the specific environmental conditions in which the experiences occurred. In other words, we could hypothesize that these experiences have emerged from specific links to the running environment, and specifically to the race topography which provides suitable conditions for their emergence [27]. A specific analysis of courses of experience in ascents, descents, and at resupply points thus appears to be relevant, because trail runners often characterized the perceived difficulties in their races in relation to this element of the topography [28].

To attain this goal and provide a detailed analysis of these races, the methodology of the course of action approach [10] was used. This methodology analyses in depth the different streams of experience that are meaningful to each person, i.e., streams that are “showable, narratable and commentable to an observer or interlocutor” and resulting from the succession of links between actions and situations (e.g., Theureau & Jeffroy, p. 19) [10]. In sports psychology, many studies [20,25,26,29] have used this framework to show how experience during a sporting activity could be thought of as a succession of discrete sequences that have an impact on that unfolding activity’s outcome.

Continuing from the above studies, the present study of trail runners aimed to characterize withdrawals in relationship to the race topography as a part of the overall running environment. We paid particular attention to the topographical characteristics of where in the race withdrawers identified different stages of their activity. This would help us to test our hypothesis about the relationship between the topographical characteristics of a particular point in the race (i.e., ascents, descents, and stops at resupply points) and runners’ emergent activities [3].

## 2. Method

### 2.1. Participants

Eight male and two female runners between 19 and 54 years old (M = 38.3; SD = 8.90), who had run in one of the three *Grand Raid de la Réunion* races on the island of Réunion volunteered to participate in this study. All were amateur athletes with experience in very long endurance races; they had run between 29.14% and 77.30% of the race distance (M = 44.54; SD = 15.43) before their withdrawal. The longest race, known as the *Diagonale des Fous*, crosses the island from the southeast to the northwest and is 172.6 km long with 9996 m of positive and negative elevation change. The middle race, called the *Bourbon Trail*, is 97 km long with 5655 m of positive and negative elevation change, and the shortest race is the *Mascareignes Trail*, 65 km long with 3922 m of positive and negative elevation change.

When they first met the researchers, participants were informed that the study aimed to examine their perceptions of how their race unfolded physically and psychologically, and that participation was completely voluntary. They were also informed that all the data and their analyses would be rendered anonymous, although they might be presented in various professional and scientific settings. Data collection, the general principles of data processing, and the research hypothesis were all explained before the runners gave their written informed consent to participate. The protocol was approved by the Research Ethics Committee of the University of Lausanne’s Faculty of Social Science and it abided by the Declaration of Helsinki.

### 2.2. Research Design

Our research was designed to process two types of data: (a) recorded and transcribed reports elicited by researchers during enactive interviews with runners who were confronted with traces of their own past activity and asked to rebuild their experience [3]; and (b) counting each type of sequences related to the topography.

### 2.3. Data Collection

Two types of data were collected in order to help build up each runner’s individual course of experience database and to reveal the seven representative sequences: (a) traces of past activity using the race map that depicted the geographic landmarks, aid stations and the elevation changes of the route; and (b) recorded and transcribed data from the self-confrontation interviews. 

Participants built up a description of their race activity—one to three days after the race—by identifying where on the race map the changes in their courses of experience had occurred. Each participant was asked to mark the topographical features on the map where their activity and courses of experience had shifted (e.g., gastric problems, cramps, feelings of unease, high speed running periods). To help them, the race map was augmented with aerial and landscape photographs identifying resupply points, the running trail itself, altitudes, and other geographical indicators within the race environment (e.g., place names or distance to the next resupply point). The self-confrontation interviews were carried out immediately afterward and lasted between 60 and 120 min. More generally, prompted interviews used in previous sports sciences research [21,30,31] were designed to collect information generated as the actions in the race story unfolded. All interviews were recorded and transcribed for further analysis. 

### 2.4. Data Processing

Data were processed in five steps (for details on points (a) to (d) see Antonini Philippe et al. [3]): (a) rebuilding the race story using elementary units of meaning (EUMs); (b) characterizing each runner’s story by grouping EUMs into meaningful sequences; (c) identifying representative sequences composed of runners’ typical stories; (d) characterizing each type of sequence leading to withdrawal in depth; and (e) counting each type of sequence with regards to the topography (ascents, descents, and resupply points). 

Rebuilding race stories using Elementary Units of Meaning (EUMs). This data processing step identified and labelled the EUMs that characterized each part of the runner’s course of experience during the race. By using the information which each participant added to the map and the stories collected during self-confrontation interviews, EUMs were identified by breaking down the runner’s experience, step-by-step, into meaningful parts that answered the following questions: What was the runner doing? What was he/she thinking? What was he/she feeling? 

Characterizing each trail runner’s story by grouping EUMs into meaningful sequences. This process step examined the coherence of relationships between EUMs. Each coherent relationship was made up of units forming a chain around the meaningful concerns forming a sequence of experience in the runner’s activity. 

Identifying the representative sequences making up runners’ typical stories. We compared the sequences described in the runners’ courses of experience in order to detect where the common structures in those sequences were situated in time. When sequences contained a common theme and were identified in every runner’s courses of actions, they were considered to be representative sequences experienced by runners and were labeled as typical sequences. 

Characterizing each typical sequence in depth. After having identified the representative sequences, we attempted to put boundaries on the diversity of their experiential content. To do this, we looked at each runner’s sequences in depth so as to characterize the different constituent parts of their courses of experience. 

Counting typical sequences with regards to topography. After having identified the typical sequences, we proceeded to associate each sequence with the race’s topography. Using the race map, the number of times each typical sequence was associated with an ascent, a descent, or a resupply point was counted. 

### 2.5. Data Reliability and Analysis

Several measures were taken to ensure the reliability of the data and their analysis. First, the researchers involved were experienced in conducting qualitative research, particularly in using the course of experience approach. Second, data were collected by experienced researchers in sport sciences and psychology, supervised by a third experienced researcher in course of experience methodology. They also underwent specific practical training on how to perform this specialised data collection and coding process. Third, data were coded independently by each of three researchers, including the researcher experienced in course of experience methodology. Coding procedure reliability was assessed using Bellack’s agreement rate and ranged from 70% to 90% between coders for the different representative sequences and the common structures between them. The inter-coder reliability was sufficiently high (i.e., higher than 0.70 [32]) to ensure the objectivity of the encoding process. When all three researchers disagreed, the data were ignored; when two of the three researchers were in agreement, they collectively re-examined the data until an agreement was reached with the third coder (no discrepancies were observed in these cases).

A Chi-square goodness of fit test with a uniform distribution using Monte Carlo simulation was used to compare the numbers of sequences, which withdrawers experienced in the different topographical areas. The Monte Carlo method uses repeated random sampling to generate simulated data to use with a mathematical model. A *p*-value of 0.05 or less was considered an acceptable level of significance.

## 3. Results

The trail runners’ courses of experience were made up of seven typical sequences (i.e., feeling pain; putting meaning to those feelings; adjusting running style; attempting to overcome the problem; the influence of other runners; assessing the situation; and deciding to stop) that lead to withdrawal [6]. The last typical sequence—deciding to stop—will not be considered in the present study because the race rules stipulate that this can only occur when a runner is at a resupply point. Table 1 shows the distribution of different sequences and where runners most associated them in relation to the race topography.

Results indicated that less than 20% of the sequences occurred during descents and most of them occurred at resupply points (46.2%) or in ascents (35.6%); this distribution was significant (*p* < 0.05). 

Significant statistical differences in the distribution of the sequences among the different categories of topography (*p* < 0.05) were observed for PM (putting meaning to those feelings), OP (attempting to overcome the problem), IR (the influence of other runners), and AS (assessing the situation). 

The results showed that there were significantly more sequences of PM in ascents (i.e., 57.2% in ascent, 17.8% in descents, and 25% at resupply points), whereas there were more sequences of OP, IR and AS at resupply points.

No significant differences in the repartition of sequences of FP (feeling pain) or AR (adjusting running style) were observed in the different categories of topography (*p* > 0.05), indicating that these two types of experience could occur independently of race topography.

Finally, even though it was not statistically significant, the descents had the most sequences of FP and AR. 

## 4. Discussion

This study used typical sequences from the courses of experience elicited from runners in an ultra-trail race [3]. Their courses of experience were discernible via seven typical sequences (i.e., feeling pain, putting meaning to those feelings, adjusting running style, attempting to overcome the problem, the influence of other runners, assessing the situation, and deciding to withdraw). Below, we discuss these results in the framework of the first six sequences and how they were linked with the topography.

The first sequence, “feeling pain” (FP), characterized by the development of a physical pain, was not linked to the race’s topography. At the stage when pain develops, the trail runners are not involved in any particular cognitive activity or strategic management of effort. They experience pain, tolerate it, and accept the resulting discomfort in ascents, descents, and at resupply stops. Thus, pain appears to be an integral or general part of trail running [9,33]. Trail runners seem to be waiting for pain to arrive; it is an expected part of the ultra-trail race experience and can develop at any moment without any particular link to the topography. In other words, pain is not set-off or worsened by the specificities of the terrain. Feeling pain is an experience embodied and embedded in the general unfolding of an ultra-trail race.

The second sequence, “putting meaning to those feelings” (PM), involved the interpretation of those feelings of pain; it was the logical next step because the trail runners tried to cognitively make sense of the symptoms of their pain, but also because the pain becomes so sharp that it must be investigated. The search for the causes of pain manifested itself differently according to the topography and mainly took place during ascents. An initial hypothesis might suppose that, strategically, this activity of trying to understand the pain should occur during descents because they are less physically demanding parts of the race [3]. In contrast, however, our results showed that ascents were where most of this sequence of activity occurred. We can thus hypothesize that ascents were situations that facilitated this cognitive process. In descents, things are happening quickly and runners must remain extremely focused in order to avoid falls [28]. Ascents required less concentration and more physical performance per se, allowing runners possibilities to develop another cognitive activity—making sense of what was happening to them. Thus, the interpretation of feelings, perceptions, and beliefs that Gammage et al. [34] observed in trail runners’ inner dialogues, and the instructions that they could “give” themselves, was mostly embedded in the race’s ascents. 

The next sequences, “adjusting running style” (AR), deals with the embodied and cognitive coping strategies that trail runners develop in order to manage situations that are starting to become difficult to handle. The adjustment of running styles took place whatever the type of topography was. It suggests that adaptations are more focused on motor skills in both ascents and descents [35]. For example, reducing speed in a descent means that the body must increase its resistance to the force produced by the interaction between the runner’s weight and the steepness of the slope, whereas reducing speed in an ascent is achieved by decreasing the intensity of the forces generating the movement [35]. Thus, even the topography is considered differently for this kind of adaptation; it offers twice as many possibilities that appear to be used in an equivalent manner by the runners [5].

“Attempting to overcome the problem” (OP) occurred mainly at resupply points. Coping in the previous sequences mainly involved motor skills; however, for this sequence, trail runners tried to manage their problems in a behavioural and in an emotional way [36]. This adaptation requires a thought process: runners must have the necessary cognitive resources to concentrate on their problem and the opportunity to put their feelings and the events of the day into perspective in order to solve that problem (e.g., What did I do? What are the solutions?). Solving a problem is thus no easy task when running. Hence, we can hypothesize that the resupply points offered a better topographical position and situation for developing this kind of cognitive activity than the running parts of the race. By carrying out this sequence activity in these places, runners enacted a situation that embedded a specific form of problem solving into a specific context (i.e., find a solution “at rest”). This transformed the resupply points into “crossroads” space and time where different organizations of their activity could be emotionally and cognitively examined and a behavior or reaction could be chosen. The resupply points are constructed as a setting for embedding this activity that opened up a possibility to accurately solve the problem and examine the possibility of managing the activity’s organization differently. These results could be linked to previous studies on sports and games; these revealed that the succession of sequences that composed an activity was embedded in a dynamic of meaningful and specific times and places for each sequence [22,31,37]. In each of these studies, these “crossroads” activities were not embedded independently of the possibility offered by the context but when and in specific time, place or succession of events makes possible the required adaptation. 

The sequence involving the “influence of other runners” (IR) corresponded to the impacts which other runners’ activities might indirectly have on their peers, such as discussions and comments. Indeed, this activity did not occur during descents, but did during ascents and mostly at the resupply points. It seems that descents require much more concentration on motor skills, as the risk of a fall is far higher, and thus the runners paid far less attention to other athletes during these parts of the race. Ascents, however, require less complicated and slower physical effort, as noted before [35]. Ascents offer the possibility to pay some attention to what others are doing and how they were running. For example, runners could be aware of receiving encouragement from others or, on the contrary, could feel discouraged when seeing faster runners overtaking them. As seen in the “attempting to overcome the problem” sequence, the resupply points were the spots where the trail runners really asked questions of themselves, discussed things with their entourage, and weighed up the situation. Finding solutions to problems could be developed in relation to other people. Resupply points gave runners the possibility to discuss with other runners, friends, family members or physiotherapists. They are thus the right place for interpersonal activities, and our results showed that activities there went beyond mundane discussions but focused on the very real problems encountered during ultra-trail races [3]. 

The sixth sequence, “assessing the situation” (AS), the last one we will examine here, was characterized by an overall and final assessment of the events that had occurred in the race so far, as well as the runner’s current state of vitality and situation in the race. Once again, this sequence mostly appeared at resupply points. It is a crucial stage in the runner’s course of experience because this is the sequence that precedes the decision to continue or withdraw from the race. Again, this activity took place more often when the trail runners had stopped in resupply points, places where they could more easily take a figurative step back from the race and think about the options available to them and/or the decisions to be made. The same analysis about the role of supply point made previously could be used for this last sequence. 

Our results showed that fast all these sequences appeared to be involved in a coping strategy process where the runners tried to better understand the discomfort caused by the difficult of the run in order to better cope with it [36].

Finally, our results showed that different activities occurring on the trail often had a significant relationship to the race topography. Notably, the resupply points, rather than steep ascents, seemed to be places on the trail where the sequences leading to withdrawal from the race occurred. This was either due to certain influences derived from other runners [3] or simply because they were the places on the trail where runners could take stock of their personal situation-moments when the path towards withdrawal was being drawn. This situation can also be related to the trail runner’s type of activity at the resupply point, i.e., rest and recuperation. Runners now have a little time and space to think, to carry out a cognitive evaluation of their overall management of their physical efforts in the race [6,8]. Hence, each sequence was related to the runner’s particular capacities for acting, feeling and thinking which were embedded in specific places along the trail-in its topography, forming a whole. This whole allowed the unfolding of the activity and increased overall effectiveness of runners’ activity in relation to the constraints of this specific sport.

Although this study revealed valuable findings, some limitations must be acknowledged. From a methodological point of view, a retrospective design creates certain difficulties. Some of our data were collected during post-race interviews, which inherently raises the question of retrospective recall, for example the memory processes. Previous studies, which also used self-confrontation interviews, have confirmed that it is possible to limit the weaknesses of traditional verbal reporting [31]. Another limitation concerns the sample. It would have been pertinent to compare our group of withdrawals with a group of finishers in order to best identify the influence of topography on these ultra-trail runners.

## 5. Conclusions

To conclude, this study allowed us to highlight important information on how ultra-trail runners manage their races in relationship to the race environment and more specifically to its topography. Our new knowledge and understanding of ultra-trail runners’ courses of experience will help us to better explain their activities. However, they will also help us to recommend potential adjustments to ultra-trail runners’ performance-oriented training and preparation; i.e., help them to finish ultra-trail races, and especially help them to learn how to deal with the sequences which lead towards withdrawal and principally manifest themselves at resupply points. Our findings also suggested the importance of the use of mental techniques in the development of mental toughness, notably through the control of emotions. Sport psychologists/mental coaches could take a more active role by explicitly helping them develop and strengthen their mental skills strategies in an adaptive and efficient way.

## Figures and Tables

**Table 1:** Comparison of the repartition of sequences by the withdrawers in the different topography settings.

Sequences	Descent		Ascent		Stop (Life Bases)		*P*
	n	%	n	%	n	%	
Feeling pain (FP)	10	31.2	14	43.8	8	25	0.4633
Putting meaning to those feelings (PM)	5	17.8	16	57.2	7	25	0.0269 *
Adjusting running style (AR)	6	40	7	46.6	2	13.4	0.3593
Attempting to overcome the problem (OP)	0	0	0	0	7	100	0.0009 *
Influence of other runners (IR)	0	0	5	23.8	16	76.2	0.0005 *
Assessing the overall situation (AS)	3	10.3	5	17.3	21	74.4	0.0005 *
Total	24	18.2	47	35.6	61	46.2	0.0003 *

Note: * Statistically significant

## References

[1] Simpson D., Young G., Jensen P.R. (2014). “It’s not about taking the easy road”: The experiences of ultramarathon runners. Sport Psychol..

[2] Runraid.free.fr 2015. La Réunion—Grand Raid Diagonale des Fous—Palmarès Complet Depuis la Lère Marche des Cimes, de 1989 à 2014. http://runraid.free.fr/grand_raid/palmares.php.

[3] Antonini Philippe R., Rochat N., Vauthier M., Hauw D. (2016). The story of withdrawals during an ultra-trail running race: A qualitative investigation of runners’ courses of experience. Sport Psychol..

[4] Hauw D., Rochat N., Gesbert V., Astolfi T., Antonini Philippe R., Mariani B., Salmon P., Macquet A.C. (2016). Putting together first- and third-person approaches for sport activity analysis: The case of ultra-trail runners’ performance analysis. Advances in Human Factors in Sports and Outdoor Recreation.

[5] Holt N.L., Lee H., Kim Y., Klein K. (2014). Exploring experiences of running an ultramarathon. Sport Psychol..

[6] Rochat N., Hauw D., Philippe R.A., von Roten F.C., Seifert L. (2017). Comparison of vitality states of finishers and withdrawers in trail running: An enactive and phenomenological perspective. PLoS ONE.

[7] Havenar J., Lochbaum M. (2007). Differences in participation motives of first-time marathon finishers and pre-race dropouts. J. Sport Behav..

[8] Johnson U., Kenttä G., Ivarsson A., Alvmyren I., Karlsson M. (2015). An ultra-runner’s experience of physical and emotional challenges during a 10-week continental run. Int. J. Sport Exerc. Psychol..

[9] Lahart I.M., Lane A.M., Hulton A., Williams K., Godfrey R., Pedlar C., Wilson M.G., Whyte G.P. (2013). Challenges in maintaining emotion regulation in a sleep and energy deprived state induced by the 4800 Km ultra-endurance bicycle race; the Race Across America (RAAM). J. Sports Sci. Med..

[10] Theureau J., Hollnagel E. (2003). Course-of-action analysis and course-of-action centered design. Handbook of Cognitive Task Design.

[11] Froese T., Di Paolo E.A. (2011). The enactive approach: Theoretical sketches from cell to society. Pragmat. Cogn..

[12] McGann M., De Jaegher H., Di Paolo E. (2013). Enaction and psychology. Rev. Gen. Psychol..

[13] Stewart J.R., Gapenne O., Di Paolo E.A. (2010). Enaction: Toward a New Paradigm for Cognitive Science.

[14] Varela F., Thompson E., Rosch E. (1991). The Embodied Mind: Cognitive Mind and Human Experience.

[15] Hauw D. (2017). Enaction et intervention en psychologie du sport chez les sportifs élites et en formation [Enaction and intervention in sports psychology for elite and in training athletes]. Can. J. Behav. Sci..

[16] Norman D.A. (1988). Psychology of Everyday Things.

[17] Thompson E. (2005). Sensorimotor subjectivity and the enactive approach to experience. Phenomenol. Cogn. Sci..

[18] Gibbs R.W. (2005). Embodiment and Cognitive Science.

[19] Theureau J., Jeffroy F. (1994). Ergonomie des Situations Informatiséesi.

[20] Hauw D., Durand M. (2004). Elite athletes’ differentiated action in trampolining: A qualitative and situated analysis of different levels of performance using retrospective interviews. Percept. Mot. Skills.

[21] Hauw D., Durand M. (2007). Situated analysis of elite trampolinists’ problems in competition using retrospective interviews. J. Sport Sci..

[22] Hauw D., Durand M. (2005). How do elite athletes interact with the environment in competition? A situated analysis of trampolinists’ activity.. Eur. Rev. Appl. Psychol..

[23] Hauw D., Renault G., Durand M. (2008). How do aerial freestyler skiers land on their feet? A situated analysis of athletes’ activity related to new forms of acrobatic performance. J. Sci. Med. Sport.

[24] Hauw D., Mohamed S. (2015). Patterns in the situated activity of substance use in the careers of elite doping athletes. Psychol. Sport Exerc..

[25] Mottet M., Saury J. (2013). Accurately locating one’s spatial position in one’s environment during a navigation task: Adaptive activity for finding or setting control flags in orienteering. Psychol. Sport Exerc..

[26] Sève C., Saury J., Ria L., Durand M. (2003). Structure of expert players’ activity during competitive interaction in table tennis. Res. Q. Exerc. Sport.

[27] Rietveld E., Kiverstein J. (2014). A rich landscape of affordances. Ecol. Psychol..

[28] El Helou N., Tafflet M., Berthelot G., Tolaini J., Marc A., Guillaume M., Hausswirth C., Toussaint J.F. (2012). Impact of environmental parameters on marathon running performance. PLoS ONE.

[29] Hauw D., Durand M. (2008). Temporal dynamics of acrobatic activity: An approach of elite athletes specious present. J. Sports Sci. Med..

[30] Briki W., den Hartigh R.J., Hauw D., Gernigon C. (2012). A qualitative exploration of the psychological contents and dynamics of momentum in sport. Int. J. Sport Psychol..

[31] Mohamed S., Favrod V., Antonini Philippe R., Hauw D. (2015). The situated management of safety during risky sport: Learning from skydivers’ courses of experience. J. Sci. Med. Sport.

[32] Van Someren M.W., Barnard Y.F., Sandberg J. (1994). The Think aloud Method: A Practical Guide to Modeling Cognitive Processes.

[33] Meyers M.C., Bourgeois A.E., LeUnes A. (2001). Pain coping response of collegiate athletes involved in high contact, high injury-potential sport. Int. J. Sports Psychol..

[34] Gammage K.L., Hardy J., Hall C.R. (2001). A description of self-talk in exercise. Psychol. Sport Exerc..

[35] Schücker L., Hagemann N., Strauss B., Völker K. (2009). The effect of attentional focus on running economy. J. Sports Sci..

[36] Lazarus R.S., Folkman S. (1984). Stress, Appraisal, and Coping.

[37] Kirsch D. (1996). Adapting the environment instead of oneself. Adapt. Behav..

